# Nervous and Mental Diseases

**Published:** 1906-01

**Authors:** R. F. Sheehan

**Affiliations:** Buffalo, N. Y.; Late House Physician at the Buffalo General Hospital


### PROGRESS IN MEDICAL SCIENCE.

### Nervous and Mental Diseases

By R. F. SHEEHAN, M. D., Buffalo, N. Y.
Late House Physician at the Buffalo General Hospital.

Myophobia, by John Punton (*Journal Nervous and Mental diseases*— October, 1905) That if there is a dividing line between the neuroses and psychoses insanity exists upon both sides of this line. Hence the term neurasthenia is often a polite misnomer for insanity.

That a large number of the so-called neurasthenias, and all the hysterias should be classed as the prodromal stages of the psychoses.

That these non-insane psychoses, however, as a rule first come under the observation of the family physician, who unfortunately often fails to recognise their true psychological significance until the obsession impulse becomes most conspicuous.

That the vast majority however, said to be suffering from these nervous effections, upon strict examination are found to be afflicted with a true psycho-neurosis, which we term psychasthenia or which better might be called psychosomatasthenia.

That because we do not recognise these conditions, as true psychoses, we neglect stringent measures of treatment and so account for our inability to cure such ailments, as the longer the duration, without appropriate treatment, the less chance of recovery.

That if agreed that insanity in its incipiency is curable, why allow it to become incurable before applying the legitimate means and measures that favor its cure.

From which the following conclusions are deduced.

1st. That the close relation which exists between the so-called neurasthenias and insanity is so very striking, as to establish a true equivalency.

2d. That curability of these insanities largely depends upon the early enforcement of appropriate medical treatment.

3d. That the non-insane psychoses are subject to the same theraputic laws and principles which apply to the more pronounced forms of incipient insanity.

4th. That their prognosis is in direct ratio to their duration.

5th. That violation, either through ignorance, pride, or wilful neglect of these fundamental essentials is the responsible agent of the rapidly increasing tide of incurable mental disorders.

ANESTHESIA ASSOCIATED WITH HYPERALGESIA SHARPLY CONFINED TO AREOLA-NIPPLE AREA OF BOTH BREASTS. A NEW AND APPARENTLY CONSTANT STIGMA IN HYSTERIA.

William W. Graves M.D., (*St. Louis Journal Nervous and Mental Diseases,* October, 1905.) Observations from a large number of cases.

1st. That in normal individuals this sensory disturbance was not found. On the contrary, the majority of these claimed to perceive touch more, and painful impressions less acutely in the areola-nipple area, than in the surrounding parts, whereas others noted little or no difference.

2d. That it was not present in any organic disease, neurosis or pspchosis, unless hysteria was a complication, and the diagnosis of such complication was made independently of it.

3d. That in every case of hysteria, thirty in all, six males and twenty-four females, including the mildest and severest examples of the malady, two of the males having traumatic hysteria, areola-nipple anesthesia associated with hyperalyesia was invariably present, confirming in a striking manner to my first observation.

Castor Oil Treatment of Trifacial Neuralgia, by George Gill, M.D., (*Cleveland Medical Journal,* August, 1905) The use of castor oil 30, in the morning one hour before breakfast, combined with the use of strychnine nitrate hypodermically, morning and evening, in increasing doses.

Its use is entirely empirical. It is difficult to be sure in what cases it will prove effective, but it should be tried with patience and persistance in all cases.

Besides its cathartic action castor oil has a well recognised quieting effect; this is often noticed when given in doses short of catharsis, where it is retained in the bowel for a time.

When given in large doses, after the first few days it appears to lose its cathartic effect, and then does not usually move the bowels more than once. Its action in neuralgia is not due to its cathartic effect because other means of catharsis tried in the same cases do little good.

ACUTE INTERIOR POLIOMYELITIS IN A YOUTH, by Theodore A. Hoch, M.D., (*Insane Hospital Journal Nervous and Mental Diseases,* October, 1905) Conclusions from a case and review of the literature upon this subject.

1st. Anterior Poliomyelitis is the result of a primary inflammatory disease of the blood vessels of the cord, which may be thrombotic or embolic.

2d. The destruction of the ganglion cells is secondary and depends in part upon the deficient blood supply of the diseased area, and in part upon pressure and toxins.

3d. The pathological changes occurring in poliomyelitis of children and adults are apparently identical and dependent upon similar causes.

4th. There is sufficient evidence at hand to consider the disas a rule of an infectious nature, however not depending upon a specific micro-organism, but resulting from bacterial infections of various kinds, and at times from other poisons.

5th. The inflammatory changes are present in the peripheral vessels as well as in the branches of the anterior spinal artery, though these changes are seldom visible until the vessels enter the gray matter.

6th. The inadequate collateral circulation within the anterior horns is favorable for sluggish circulation and embolism.

---

BRACHIAL BIRTH PALSY. Conclusions regarding its etiology and pathology, symptomatology and treatment. L. P. Clark, M.D., A. S. Taylor, M.D., T. P. Trout. (*American Journal of the Medical Sciences,* October, 1905.)

1. The cause of the laceration type of birth palsy is tension on the nerve trunks, which first ruptures the nerve sheath, then the nerve fibres. The prevention of this serious lesion of the cervical nerve trunks rests with the obstetrician, who should not over stretch the child's neck in the process of delivery.

2. The persistence of the palsy is clearly explained by the pathological findings, viz: (a.) Rupture of the perineural sheath with hemorrhage into its substance, resulting in the formation of hematomata or hematomatous infiltration into the surrounding tissues.

(b.) The cicatrical contraction following organisation of the blood clot and repair of the rent in the perineural sheath. The connective tissue thus formed indents and presses upon the nerve bundles, strangulating them, and preventing regeneration of the nerve fibres. In some instances the same result is accomplished by the turning inward of the perinenial sheath upon the nerve bundles.

3. The nature of the lesion in all cases demands excision of the damaged areas and suture of the divided ends as soon as it is proven that spontaneous repair will not take place. The plan of treatment is then the same as for peripheral nerve injuries elsewhere.

4. In all cases such treatment as will prevent contractures and deformities and maintain muscle tone in the paralysed limb should be systematically used until either spontaneous recovery occurs or operation is done. (Traumatic neuritis is a contraindication to active treatment.) It is obvious that the above measures should be continued after operation.

5. The proper time for surgical intervention is not yet definitely fixed. It appears, however, to much later than two or three months after birth, as advised in Kennedy's report. At the present time one year would seem to be a reasonable delay before operation.

6. Sufficient time has not elapsed in the majority of cases in this series for final results to have appeared.

At the end of eighteen months in two cases, the improvement in nutrition, range of motion, and muscle power in the paralysed limb have been sufficient to demonstrate the value of the operative procedure.

---

General Paralysis and Tabes Dorsalis, by Henry A. Cotton (*Am. Jour. Insan.,* April, 1905)

Conclusions upon coincidence.—

1st. That, clinically, tabes and general paralysis present many analogies in eitology, symptomatology and course.

2d. That their occurence in the same individual is more than coincidence.

3d. That in these cases of tabo paralysis the symptoms presented are identical with the symptoms of general paralysis and tabes when seen apart, only differing in degree, according to the extent of the anatomical lesion.

4th. That the clinical symptoms of tabo-paralysis have the same anatomical basis as in the separate diseases.

5th. That anatomically the affection of the posterior columns if the cord, as seen in tabo-paralysis, does not differ from the picture presented in pure tabes.

The same symptoms are affected, and the segmental character of the process is the same, also that the process in the cortex is identical with that of general paralysis.

6th. While the above facts show the intimate relation between general paralysis and tabes dorsolis, the unsettled status of their pathogenesis at present prevents their identity being absolutely established upon an anatomical basis.

